# Update to Interim Guidance for Preexposure Prophylaxis (PrEP) for the Prevention of HIV Infection: PrEP for Injecting Drug Users

**Published:** 2013-06-14

**Authors:** Dawn K. Smith, Michael Martin, Amy Lansky, Jonathan Mermin, Kachit Choopanya

**Affiliations:** Div of HIV/AIDS Prevention, National Center for HIV/AIDS, Viral Hepatitis, STD, and TB Prevention, CDC; Bangkok Tenofovir Study Group, Thailand

On June 12, 2013, the Thailand Ministry of Health and CDC published results from a randomized controlled trial of a daily oral dose of 300 mg of tenofovir disoproxil fumarate (TDF) that showed efficacy in reducing the acquisition of human immunodeficiency virus (HIV) infection among injecting drug users (IDUs) (1). Based on these findings, CDC recommends that preexposure prophylaxis (PrEP) be considered as one of several prevention options for persons at very high risk for HIV acquisition through the injection of illicit drugs.

## Background

Among the approximately 50,000 new HIV infections acquired each year in the United States, 8% were attributed to injection-drug use in 2010 (2). The National HIV Behavioral Surveillance System, surveying IDUs in 20 U.S. cities in 2009, found high frequencies of both injection-drug use and sexual practices that are associated with HIV acquisition (3). Among IDUs without HIV infection, 34% reported having shared syringes in the preceding 12 months, and 58% reported having shared injection equipment; 69% reported having unprotected vaginal sex and 23% reported having unprotected male-female anal sex. Among HIV-uninfected male IDUs, 7% reported previous male-male anal sex, and 5% reported unprotected male-male anal sex. However, only 19% of male and female IDUs reported participating in an intervention to reduce risk behaviors. These findings underscore a need to provide effective interventions to further reduce HIV infections among IDUs in the United States.

Several clinical trials have demonstrated safety and efficacy of daily oral antiretroviral PrEP for the prevention of HIV acquisition among men who have sex with men (MSM) (4) and heterosexually active men and women (5,6), although two trials were unable to show efficacy, likely because of low adherence (7,8) (Table). CDC previously has issued interim guidance for PrEP use with MSM (9) and heterosexually active adults (10) and now provides interim guidance for PrEP use in IDUs.

During 2009–2013, CDC convened workgroup meetings and consulted with external subject matter experts, including clinicians, epidemiologists, academic researchers, health department policy and program staff members, community representatives, and HIV and substance abuse subject matter experts at federal health agencies, to 1) review the results of PrEP trials and other data as they became available and 2) deliberate and recommend content for interim guidance and comprehensive U.S. Public Health Service guidelines for PrEP use in the United States. The expert opinions from the IDU workgroup and other workgroups were used to develop this interim guidance on PrEP use with IDUs.

## Rationale and Evidence

The Bangkok Tenofovir Study enrolled HIV-uninfected persons who reported injecting illicit drugs in the prior year into a phase-III randomized, double-blind, placebo-controlled trial to determine the safety and efficacy of daily oral TDF to reduce the risk for HIV acquisition. In all, 2,413 eligible, consenting men and women aged 20–60 years were randomized to receive either daily oral doses of 300 mg of TDF (n = 1,204) or a placebo tablet (n = 1,209). Participants could elect to receive tablets daily by directly observed therapy or receive a 28-day supply of daily doses to take home; they could switch medication supply method at their monthly follow-up visits. At follow-up visits every 28 days, individualized adherence and risk-reduction counseling, HIV testing, pregnancy testing for women, and assessment for adverse events were conducted. An audio computer-assisted self-interview was conducted every 3 months to assess risk behaviors. Blood was collected at enrollment; months 1, 2, and 3; and then every 3 months for laboratory testing to screen for adverse reactions to the medication. At study clinics (operated by the Bangkok Metropolitan Administration), social services, primary medical care, methadone, condoms, and bleach (for cleaning injection equipment) were provided free of charge.

The study was conducted during 2005–2012, with a mean follow-up time of 4.6 years (maximum: 6.9 years) and a 24% loss to follow-up or voluntary withdrawal in the TDF group and a 23% loss in the placebo group. Participants took their study drug an average of 83.8% of days and were on directly observed therapy 86.9% of the time.

After enrollment, 50 patients acquired HIV infection: 17 in the TDF group and 33 in the placebo group. In the modified “intent-to-treat” analysis (excluding two participants later found to have been HIV-infected at enrollment), HIV incidence was 0.35 per 100 person-years in the TDF group and 0.68 per 100 person-years in the placebo group, representing a 48.9% reduction in HIV incidence (95% confidence interval [CI] = 9.6%–72.2%). Among those in an unmatched case-control study that included the 50 persons with incident HIV infection (case-patients) and 282 HIV-uninfected participants from four clinics (controls), detection of tenofovir in plasma was associated with a 70% reduction in the risk for HIV infection (CI = 2.3%–90.6%).

The rates of adverse events, serious adverse events, deaths, grade 3–4 laboratory abnormalities, and elevated serum creatinine did not differ significantly between the two groups. Reports of nausea and vomiting were higher in the TDF group than the placebo group in the first 2 months of medication use but not thereafter. No HIV infections with mutations associated with TDF resistance were identified among HIV-infected participants.

Comparing rates at enrollment with rates at 12 months of follow-up, risk behaviors decreased significantly for injecting drugs (from 62.7% to 22.7%), sharing needles (18.1% to 2.3%), and reporting multiple sexual partners (21.7% to 11.0%), and these risk behaviors remained below baseline throughout the entire period of the trial (all three comparisons, p<0.001). Rates were similar in the TDF and placebo groups.

## PrEP Recommendation for IDUs

On July 16, 2012, based on the results of trials in MSM and heterosexually active women and men, the Food and Drug Administration approved a label indication for the use of the fixed dose combination of TDF 300 mg and emtricitabine (FTC) 200 mg (Truvada) as PrEP against sexual HIV acquisition by MSM and heterosexually active women and men (11). These trials did not evaluate safety and efficacy among injecting-drug users.

CDC recommends that daily TDF/FTC be the preferred PrEP regimen for IDUs for the following reasons: 1) TDF/FTC contains the same dose of TDF (300 mg) proven effective for IDUs, 2) TDF/FTC showed no additional toxicities compared with TDF alone in PrEP trials that have provided both regimens, 3) IDUs also are at risk for sexual HIV acquisition for which TDF/FTC is indicated, and 4) TDF/FTC has an approved label indication for PrEP to prevent sexual HIV acquisition in the United States. Its use to prevent parenteral HIV acquisition in those without sexual acquisition risk is currently an “off-label” use. Reported injection practices that place persons at very high risk for HIV acquisition include sharing of injection equipment, injecting one or more times a day, and injection of cocaine or methamphetamine. CDC recommends that prevention services provided for IDUs receiving PrEP include those targeting both injection and sexual risk behaviors (12).

In all populations, PrEP use 1) is contraindicated in persons with unknown or positive HIV status or with an estimated creatinine clearance <60 mL/min, 2) should be targeted to adults at very high risk for HIV acquisition, 3) should be delivered as part of a comprehensive set of prevention services, and 4) should be accompanied by quarterly monitoring of HIV status, pregnancy status, side effects, medication adherence, and risk behaviors, as outlined in previous interim guidance (9,10). Adherence to daily PrEP is critical to reduce the risk for HIV acquisition, and achieving high adherence was difficult for many participants in PrEP clinical trials (Table).

## Comment

Providing PrEP to IDUs at very high risk for HIV acquisition could contribute to the reduction of HIV incidence in the United States. In addition, if PrEP delivery is integrated with prevention and clinical care for the additional health concerns faced by IDUs (e.g., hepatitis B and C infection, abscesses, and overdose), substance abuse treatment and behavioral health care, and social services, PrEP will contribute additional benefits to a population with multiple life-threatening physical, mental, and social health challenges (12,13). CDC, in collaboration with other federal agencies, is preparing comprehensive U.S. Public Health Service guidelines on the use of PrEP with MSM, heterosexually active men and women, and IDUs, currently scheduled for release in 2013.

## Figures and Tables

**TABLE:** Results from randomized, placebo-controlled, clinical trials of the efficacy of daily oral antiretroviral preexposure prophylaxis (PrEP) for preventing human immunodeficiency virus (HIV) infection

Clinical trial	Participants	Type of medication	mITT efficacy*		Adherence-adjusted efficacy based on TDF detection in blood	
	
			%	(95% CI)	%	(95% CI)
Bangkok Tenofovir Study	Injecting drug users	TDF	49	(10–72)	70	(2–91)
Partners PrEP	HIV discordant couples	TDF	67	(44–81)	86	(67–94)
		TDF/FTC	75	(55–87)	90	(58–98)
TDF2	Heterosexually active men and women	TDF/FTC	62	(22–83)	84	NS
iPrEx	Men who have sex with men	TDF/FTC	42	(18–60)	92	(40–99)
Fem-PrEP	Heterosexually active women	TDF/FTC	NS	—	NA	—
VOICE	Heterosexually active women	TDF	NS	—	NA	—
		TDF/FTC	NS	—	NA	—

**Abbreviations:** mITT = modified intent to treat analysis, excluding persons determined to have had HIV infection at enrollment; CI = confidence interval; TDF = tenofovir disoproxil fumarate; FTC = emtricitabine; NS = not statistically significant; NA = data not available.

* % reduction in acquisition of HIV infection.
